# Particular findings on lung CT in patients undergoing immunotherapy for bronchogenic carcinoma

**DOI:** 10.1007/s00508-020-01667-0

**Published:** 2020-05-20

**Authors:** Lucian Beer, Maximilian Hochmair, Daria Kifjak, Alexander R. Haug, Florian Prayer, Marius E. Mayerhoefer, Christian Herold, Helmut Prosch

**Affiliations:** 1 Department of Biomedical Imaging and Image-guided Therapy, Medical University of Vienna, Waehringer Guertel 18–20, 1090 Vienna, Austria; 2 Department of Radiology and Cancer Research, UK Cambridge Center, CB2 0QQ Cambridge, UK; 3 Department of Internal Medicine and Pneumology, Krankenhaus Nord—Klinik Floridsdorf, Vienna, Austria

**Keywords:** Non-small cell lung cancer, Nivolumab, Radiology, Oncology, PD1 receptor

## Abstract

**Background:**

Immune checkpoint inhibitors have become a valuable tool in the therapeutic strategy against metastasized non-small cell lung cancer (NSCLC) as they represent an effective and safe treatment option for many patients; however, the treatment response and side effects of this class of drugs can considerably differ compared to classical chemotherapeutics. The aim of this study was to highlight specific radiological pulmonary findings of NSCLC patients treated with immune checkpoint inhibitors.

**Methods and results:**

Medical records and images of prospectively collected data from 70 patients with advanced NSCLC, treated with immune checkpoint inhibitors, were reviewed. Of the patients two experienced an initial increase in tumor size, followed by a decrease in tumor size that was described as pseudoprogression. Another patient developed a sarcoid-like reaction accompanied by clinical improvements and radiological treatment response. A further two patients developed immune checkpoint-associated pulmonary injury that was clinically and radiologically classified as pneumonitis, which responded well to anti-inflammatory treatment.

**Conclusion:**

Management of patients with NSCLC using immune checkpoint inhibitors requires a knowledge of specific clinical and radiological findings. Both oncologists and radiologists have to be aware of the most common types, including atypical response patterns, such as a sarcoid-like reaction and pseudoprogression as well as of the pulmonary side effects that can encompass pneumonitis.

## Introduction

Immune checkpoint inhibitors have revolutionized treatment for many cancer patients and enabled tremendous achievements in treatment. In 2015, the first immune checkpoint inhibitor, nivolumab, a targeted programmed death 1 (PD-1)-mediated inhibitor, was approved for patients with advanced non-small cell lung cancer (NSCLC) [1, 2]. Since then, a wealth of immune checkpoint inhibitors have joined nivolumab, including pembrolizumab, atezolizumab, and durvalumab, and have proven their efficacy in NSCLC patients. In the near future, immune checkpoint inhibitors will be used not only in an adjuvant setting, but also in a neoadjuvant setting [3].

As the number of patients receiving immune checkpoint inhibitors will considerably increase, all members of the clinical team should be familiar with the important clinical and radiological characteristics of this class of drugs. Special attention must be paid to the assessment of treatment response as well as to the identification and management of treatment-related adverse events.

Treatment response in patients with immune checkpoint inhibitors can be challenging as four different forms have been observed. Treatment response can manifest as a classical shrinkage in tumor size as well as an initial increase in tumor size and/or the development of a new lesion that shows a stable course during the continuation of treatment. Whereas the former scenario is not challenging for either clinicians or radiologists, the latter can be easily misinterpreted as treatment failure [4]. Based on the initial results of data from 487 patients with advanced melanoma treated with the PD‑1 immune checkpoint inhibitor ipililumab, so-called immune-related response criteria have been developed [5]. The phenomenon of an initial increase in the tumor burden and/or the appearance of a new lesion followed by a decrease in tumor burden has been described as pseudoprogression [6]. Infiltrating immune cells that target cancer cells are believed to be responsible for the increase in tumor volume [7]; however, pseudoprogression is a relatively uncommon phenomenon, with an incidence of only 0.6–9% of NSCLC patients [2, 8–10] and, therefore, in the majority of patients progression of tumor burden following immune checkpoint inhibitor therapy is associated with treatment failure. Importantly, the frequency of findings in line with pseudoprogression is higher during the early treatment phase 4–6 weeks after treatment initiation, than at later time points when the incidence decreases [9].

Sarcoid-like reactions are a further type of atypical response pattern that are characterized by the infiltration of lymphocytes, mainly in lymph nodes but not exclusively, as granulomatous lymphocytes have also been described in other tissues and organs, such as the bone marrow [11–13]. It has been proposed that a sarcoid-like reaction is a sign of treatment response [11]. Correct classification can be challenging, especially in the setting of NSCLC, as its appearance can mimic disease progression, and therefore, a precise clinical and radiological assessment is needed for treatment decision-making.

Pneumonitis is considered to be the most important immune-related adverse event in NSCLC patients [14]. According to a meta-analysis with 4413 patients from 8 randomized clinical trials, the incidence of all grades of pneumonitis was 3% of which 50% were high-grade clinically relevant cases. Pneumonitis was the most common cause of treatment-related deaths (4 of 2272 patients with high-grade cases) [14] and is the most therapy-limiting side effect. The symptoms are usually unspecific and up to one third of patients have no symptoms. The radiological changes associated with pneumonitis are unspecific and range from subtle ground-glass opacities to large consolidations that present as organizing pneumonia or non-specific interstitial pneumonia.

This article outlines the radiological findings of five patients with NSLC who were treated with immune checkpoint inhibitors, and who experienced either an atypical treatment response or a pulmonary immune-related adverse event.

## Material and methods

This study was approved by the local ethics committee (EK: 1521/2015) and was performed in accordance with the guidelines of the Declaration of Helsinki. All patients gave written, informed consent prior to 2‑[^18^F]fluoro-2-deoxy-D-glucose ([^18^F]FDG) PET/CT examinations. Of the five patients reported in this manuscript three were also included in a previous manuscript [15]. Patients with an atypical response pattern or radiologically detectable immune-mediated adverse events were retrospectively identified based on a prospectively collected group of 70 NSCLC patients under PD1 or PD-1L therapy between 2016 and 2019. Demographic and clinical data, including patient age, sex, and previous therapies, were obtained from the institutional database (Table 1).

**Table 1:** Patient information

Histology	Previous treatment	Checkpoint inhibitor	Special finding	Onset after start of immunotherapy	Clinical findings	Imaging	Treatment
Adenocarcinoma	S, CHT	Nivolumab	Pseudoprogression	4 weeks	Unremarkable	>20% increase SLD	N/A
Adenocarcinoma	CHT, RT	Durvalumab	Pseudoprogression	4 weeks	Unremarkable	New bone mass	N/A
Adenocarcinoma	CHT	Nivolumab	Sarcoid-like reaction	4 weeks	Dyspnea—due do pleural effusion	Pulmonary micronodule in perilymphatic distribution; mediastinal lymphadenopathy	N/A
Adenocarcinoma	CHT	Atezolizumab	Pneumonitis	4 weeks	Increasing dyspnea	Ground-glass opacities and consolidation, mediastinal lymphadenopathy	Cortisone; pause atezolizumab
Adenocarcinoma	CHT, RT	Durvalumab	Pneumonitis	11 weeks	Worsening general condition	Ground-glass opacities	Cortisone; pause durvalumab

*S* surgery, *CHT* cytotoxic chemotherapy, *RT* radiotherapy, *SLD* sum of the longest diameters, *N/A* not applicable

### Imaging

All [^18^F]FDG PET/CT examinations were performed with the same standard clinical scanning protocol using a 64-row multidetector hybrid PET/CT device (Biograph TruePoint 64; Siemens Healthineers, Erlangen, Germany) as described previously [15]. Briefly, patients were instructed to fast for at least 6h before [^18^F]FDG PET/CT. Serum glucose levels had to be less than 180 mg/dL. The PET scanner provided an axial field of view (FOV) of 216 mm, a sensitivity of 7.6 cps/kBq, and a transaxial resolution of 4–5 mm. The PET was performed 75–110 min after the intravenous administration of up to 400 MBq [^18^F]FDG. We used a 4min/bed position, with 4 iterations, and 21 subsets. The slice thickness was 5mm with a 168 × 168 matrix. We acquired a venous phase CT of the brain, thorax, abdomen, and pelvis after the injection of 100 mL of tri-iodinated, nonionic contrast medium. A tube current of 120 mA, a tube voltage of 130 kV, a collimation of 64 × 0.6 mm, a 5mm slice thickness with a 3mm increment, and a 512 × 512 matrix were applied. Patients were instructed to use shallow breathing during the image acquisition. The CT of the thorax was not acquired at full inspiration and, therefore, the diagnostic evaluation of the lung parenchyma was slightly impaired.

## Results

### Pseudoprogression

**Fig. 1:**
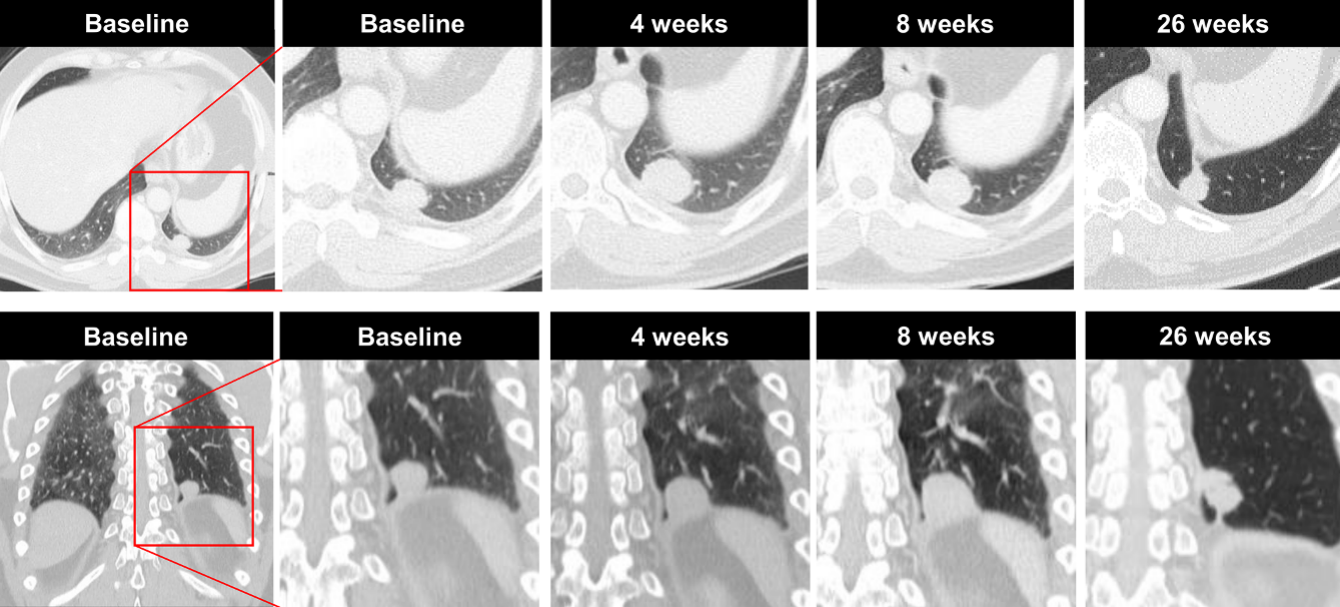
A case of pseudoprogression in a 49-year-old man with NSCLC who was receiving immune checkpoint inhibitor therapy. Coronary CT imaging obtained before therapy demonstrates a 20 mm metastasis in the left lower lobe and 4 weeks after treatment initiation, the tumor size increased from 20 to 29 mm. Therapy was continued, and 4 weeks thereafter, the tumour size was reduced by 4 mm to 25 mm. Treatment was continued and 26 weeks after treatment initiation, the tumor continued to reduce in size, measuring 14 mm. No further metastases were visible at any time point. Upper row: axial view, lower row: cornonal view

Of the patients two experienced a pseudoprogression. The first was a 47-year-old male patient who was diagnosed with an adenocarcinoma of the right upper lobe of the lung. He initially underwent lobectomy, and later developed distant metastasis and received chemotherapy (cisplatin/pemetrexed). During subsequent chemotherapy he progressed and therefore, nivolumab (3 mg/kg every 2 weeks) therapy was initiated. Before treatment, one single pulmonary metastasis was measurable, which had increased from 20 mm to 29 mm (>20%) and was classified as progressive disease according to the response evaluation criteria in solid tumors (RECIST) 11 ([16]; Fig. 1). After 4 weeks the tumor diameter remained mainly unchanged (25 mm). Except for this pulmonary metastasis, no further metastases were visible. Because of the patient’s clinical performance, the referring physician continued therapy and 26 weeks after treatment initiation, the tumor was smaller (14 mm) and remained unchanged until week 46 after treatment, at which time the tumor progressed.

**Fig. 2:**
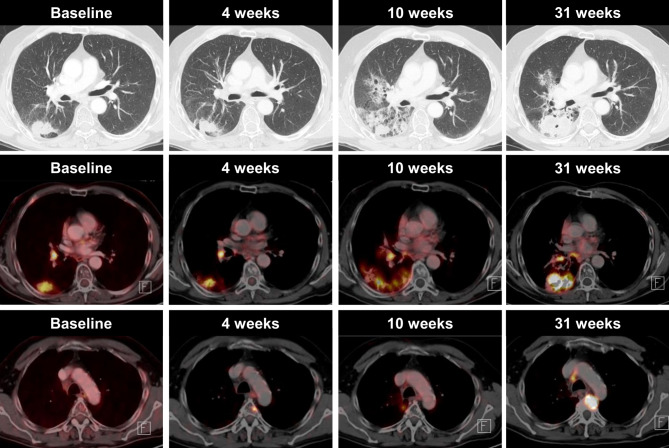
A case of pseudoprogression in a 52-year-old man with NSLCL, who was receiving immune checkpoint inhibitor therapy. Axial CT and ^18^F‑FDG-PET/CT imaging obtained before the start of treatment show a metabolically active tumor in the right lower lobe and an ipsilateral hilar lymph node metastasis. Four weeks after treatment initiation, the tumor slightly decreased in size and showed reduced ^18^F‑FDG uptake. However, we observed a newly developed, metabolically active metastasis in the 4th thoracic vertebral body measuring <1.0 cm. Treatment was continued and PET-CT performed 10 weeks after the start of treatment showed no increased ^18^F‑FDG uptake in the 4th thoracic vertebral body. The ^18^F‑FDG uptake in the primary tumour in the right lower lobe further decreased. The newly developed, metabolically active, ground-glass opacities were radiation-associated. Thirty-one weeks after the start of treatment, ^18^F‑FDG PET-CT evidenced disease progression in both the primary tumour and the metastasis in the 4th thoracic vertebral body. Upper row: CT in lung window, middle row: axial fused PET/CT image lower row: axial fused PET/CT image

The second patient was a 52-year-old male patient with an adenocarcinoma of the right lower lobe, with positive ipsilateral mediastinal (N2) lymph nodes (stage IIIA). After completion of chemoradiotherapy, he was started on durvalumab treatment (Fig. 2) and 4 weeks after the start of durvalumab treatment, a new, focal FDG uptake was visible in the thoracic vertebral body IV, while it was morphologically not visible on the CT. The primary tumor, as well as the lymph node metastasis, were unchanged and showed unchanged high FDG uptake. Treatment was continued and, on the PET/CT at 10 weeks after the start of treatment, did not show any increased FDG uptake. In contrast, CT showed a 10 × 10 mm sclerotic lesion. This was classified as pseudoprogression. The primary tumor, as well as the lymph node metastasis, slightly decreased in size and the surrounding lung parenchyma showed typical radiation-induced changes. Durvalumab treatment was continued and the disease progressed 31 weeks after treatment initiation not only on the primary tumor side and lymph node metastasis, but also on the bone metastasis on the thoracic vertebral body IV.

### Sarcoid-like reaction and pneumonitis

**Fig. 3:**
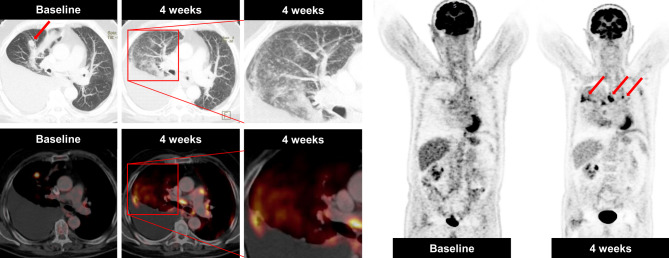
A case of a sarcoid-like reaction in a 76-year-old male NSCLC patient, who was receiving immune checkpoint inhibitor therapy. Axial CT in lung window and fused ^18^F‑FDG-PET/CT imaging obtained before treatment initiation showed a metabolically active metastasis in the middle lobe and a right pleural effusion. Four weeks after treatment initiation, multiple intrapulmonary micronodules in a perilymphatic distribution were detectable in both lungs, predominately right ≫ left. In addition, enlarged metabolically active hilar/mediastinal lymph nodes were visible (*arrows*), consistent with a sarcoid-like reaction. The disappearance of the metastasis in the middle lobe was suggestive of treatment response

A sarcoid-like reaction was observed in a 76-year-old male patient with adenocarcinoma of the middle lobe of the lung (stage IV) who received standard chemotherapy (cisplatin/pemetrexed) followed by pemetrexed maintenance therapy. At the baseline scan, he had a metabolically active metastasis in the middle lobe and a malignant pleural effusion on the right side (Fig. 3). The metastasis in the middle lobe was no longer detectable 4 weeks after start of nivolumab treatment (3 mg/kg every 2 weeks); however, he presented with multiple, newly developed, sharply delimited metabolically active nodules ordered alongside the fissures, pleura, and secondary pulmonary lobe in both lungs (right ≫ left). In addition, he developed metabolically active mediastinal lymphadenopathy. Due to the patient’s good clinical performance status and the absence of elevated infection parameters, the patient continued therapy. A further PET/CT follow-up 12 weeks after the initiation of treatment showed no changes compared to the first follow-up. Cytologic analysis of an intermittently performed puncture of the right pleural effusion remained unremarkable, with no evidence of any malignant cells. After 16 months, the patient is still continuing treatment without radiological evidence of disease recurrence.

### Pneumonitis

**Fig. 4:**
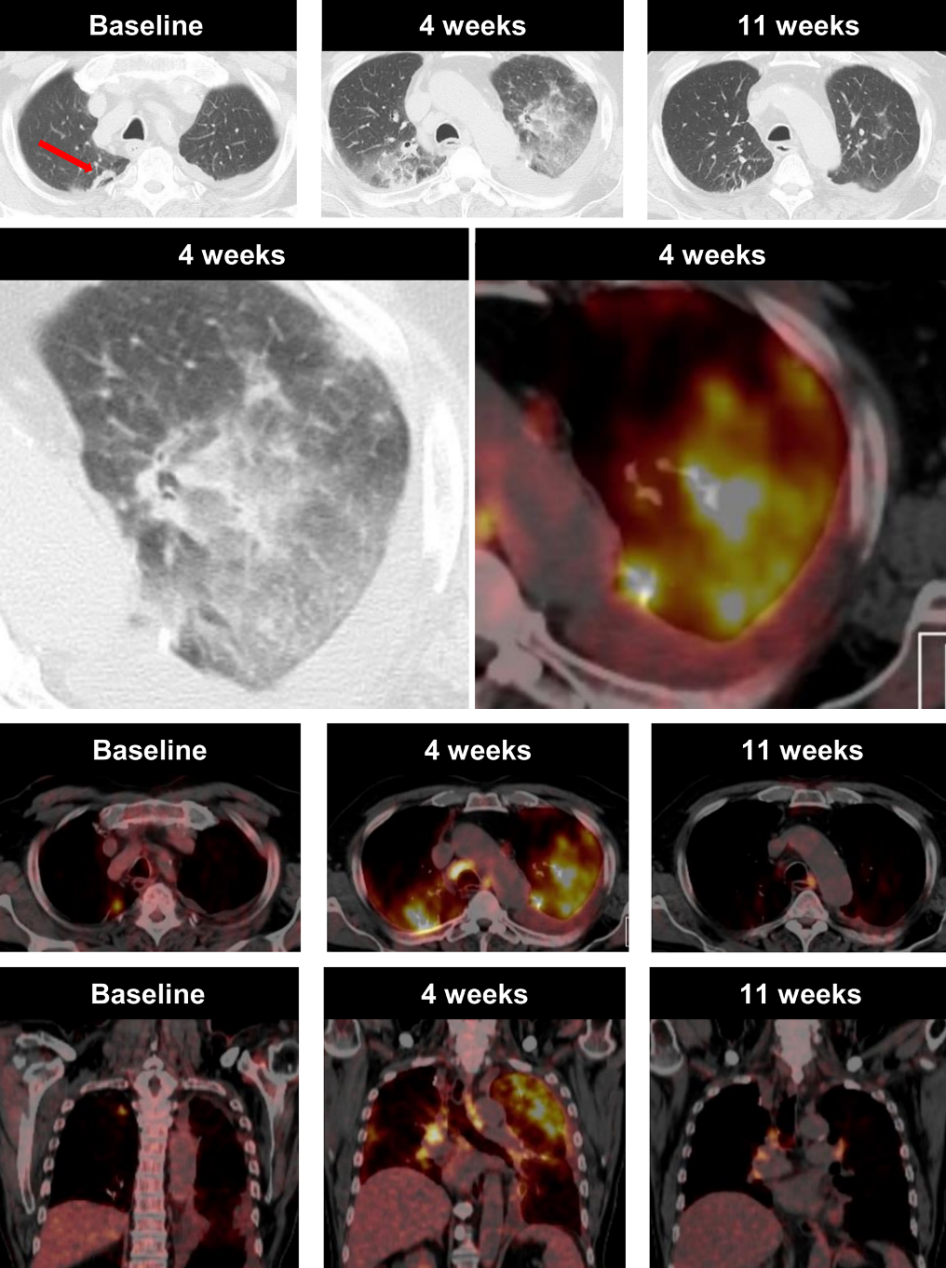
A case of pneumonitis in a 75-year-old male NSLCL patient, who was receiving immune checkpoint inhibitor therapy. Axial and coronal CT in lung window and fused ^18^F‑FDG-PET/CT imaging obtained before treatment initiation show a metabolically active metastasis in the right upper lobe, as well as five cerebral metastases (not shown). Four weeks after treatment initiation, the pulmonary, as well as the cerebral metastasis (not shown), showed a significant tumour shrinkage. In contrast, new, patchy, highly metabolically active ground-glass opacities in both lungs (upper lobes > lower lobes), as well as metabolically active enlarged mediastinal lymph nodes, were visible. On the day of the examination the treatment was discontinued and the patient was started on cortisone. After 7 weeks the pulmonary changes resolved almost completely while slightly enlarged metabolically active mediastinal lymph nodes remained. Upper three rows: axial view; lower row: coronal view

Of the patients two developed pneumonitis during the course of treatment. The first was a 75-year-old male patient with stage IV disease. At the start of treatment there was a single metabolically active pulmonary metastasis in the right upper lobe and five cerebral metastases. Four weeks after the start of atezolizumab, the patient developed increasing shortness of breath and worsened general condition. The PET/CT showed considerable newly developed ground-glass opacities in both lungs (upper lobes > lower lobes), with increased glucose activity, as well as metabolically active mediastinal lymphadenopathy (Fig. 4). At the same time, both the cerebral metastasis, as well as the lung metastasis, showed a shrinkage in size and a reduction in FDG uptake. The patient was examined by the referring physician immediately after the PET/CT. Due to normal sepsis parameters and leukocytes, cortisone was started. Consequently, the patient’s general condition improved. We performed a second follow-up examination 7 weeks thereafter, which showed a further decrease in the size of the cerebral metastasis and a complete disappearance of the lung metastasis. The ground-glass opacities resolved almost completely, as well as the mediastinal lymphadenopathy.

**Fig. 5:**
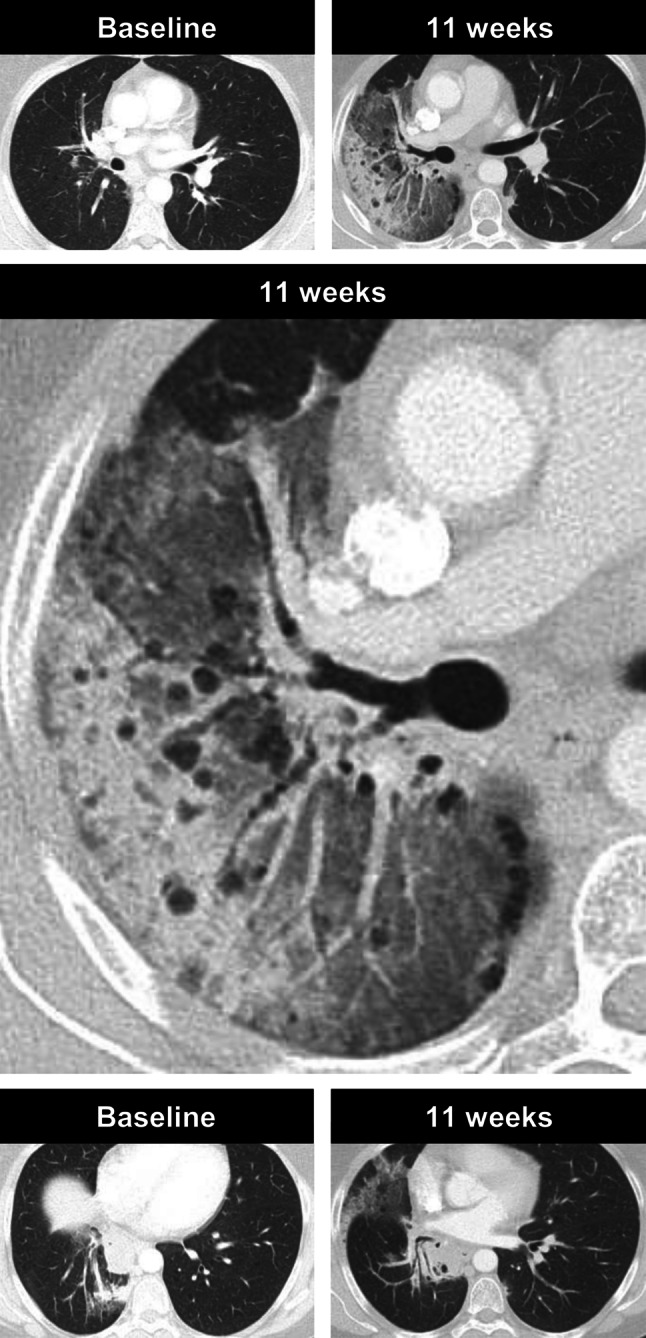
A case of pneumonitis in a 52-year-old female NSLCL patient, who was receiving immune checkpoint inhibitor therapy. Axial CT images in lung window obtained before treatment initiation show no evidence of malignant disease and 11 weeks after treatment initiation, she developed considerable ground-glass opacities and consolidations in the right upper and middle lobe without pleural effusion or signs of lymphangiosis carcinomatosa. Treatment was paused and the patient was started on cortisone. Thereafter, her general condition improved rapidly

The second patient was a 52-year-old female patient who developed severe pneumonitis during durvalumab treatment. Prior to immune checkpoint inhibitor therapy, she had sequential chemotherapy (cisplatin/pemetrexed) and radiotherapy (60 Gy) of the right hilum/mediastinum and 2 weeks after the end of chemotherapy, the immune checkpoint inhibitor therapy was started. During treatment, the patient’s general condition worsened and 11 weeks after the start of treatment, CT showed newly developed, pronounced, ground-glass opacities and smaller consolidations in the right upper lobe and middle lobe, which were not related to the radiation field (Fig. 5). Durvalumab treatment was paused and cortisone treatment started. Thereupon, her clinical performance status improved rapidly. Durvalumab was paused for 8 weeks before the patient returned to receive regular treatment doses again.

## Discussion

In this study atypical pulmonary findings in NSCLC patients in response to immune-checkpoint inhibitors were described. We showed two patients with pseudoprogression, both of whom were associated with medium-term treatment response. In addition, we presented a patient with a sarcoid-like reaction and two patients with pneumonitis, both of which can mimic a variety of different diseases.

The approval of immune checkpoint inhibitors has led to a paradigm change in the treatment of NSCLC patients; however, only ~20% of heavily pretreated NSCLC patients respond to immune checkpoint inhibitor therapy [1, 2]. Therefore, precise response evaluation as well as the detection and management of treatment-related adverse events, is of the utmost importance. Recent trials have demonstrated the diagnostic limitations of the commonly used RECIST in patients treated with immune checkpoint inhibitors. Considerable efforts have been undertaken to develop special response criteria, taking into account the particular treatment response patterns observed with immunotherapy. Several modified criteria have been published in the last few years, with the iRECIST criteria published in 2017 the most commonly used [17]. The iRECIST introduced two new response categories, namely, immune unconfirmed progressive disease (iUPD) and immune confirmed progressive disease (iCPD). In contrast to RECIST 1.1, an increase in tumor size or the development of new tumor manifestations must be confirmed by a second image examination performed 4–8 weeks after the first examination. In the interim, the category iUPD is used to classify the treatment response. If there is a further increase in tumor size or the appearance of new lesions at the second scan, the category iCPD is used to classify treatment response [17].

Pseudoprogression is the most commonly discussed atypical response pattern in patients who receive immune checkpoint inhibitor therapy. Although the laboratory [17] and radiological [7] manifestations of pseudoprogression have been described, there is no clinically used test to date that can differentiate between pseudoprogression and true progression with satisfactory certainty. The iRECIST aims to avoid the misclassification of pseudoprogression by introducing the concept of iUPD, which needs a confirmation after 4–8 weeks in order to classify iCPD. Initial progression in patients with pseudoprogression occurs most commonly within the first month after the initiation of treatment [18]. While it was initially believed that a follow-up between 4–8 weeks was suitable to confirm or rule out pseudoprogression [5], it has been recognized that lesions can remain enlarged over months even though they show a biological response [8]. From a clinical perspective, it is important to differentiate between true progression and pseudoprogression as early as possible, as the majority of patients do not benefit from treatment past defined disease progression [19]. In addition, increasing evidence shows that the response rate to chemotherapy is increased after immune checkpoint inhibitor therapy [20]. Correct identification of disease progression would enable a switch from immune checkpoint inhibitor therapy to subsequent chemotherapy.

The second form of atypical response pattern discussed in this manuscript is a sarcoid-like reaction. This might be biologically linked to the phenomenon of pseudoprogression, as both are believed to rely on the infiltration by immune cells of immune-active areas. A sarcoid-like response pattern has been associated with beneficial courses of disease in melanoma patients [11], and therefore, should not be misinterpreted as disease progression or pseudoprogression. In particular, when there are signs of treatment response (e.g., a reduction in size and glucose metabolism), the development of newly enlarged metabolically active lymph nodes (even intra-abdominal), and lung nodules in a perilymphatic distribution, are suggestive of a sarcoid-like reaction.

Pneumonitis is the most dangerous adverse event in NSCLC patients [14], with several reports of a fatal outcome [21]. As pneumonitis does not have a uniform radiological appearance, even slight signs, such as ground-glass opacities, atypical consolidations, and/or reticulations, should be reported as suspicious, especially in cases of clinical deterioration. All imaging findings in pneumonitis are non-specific and cannot be differentiated from infectious pneumonia. Any radiological signs suggestive of pneumonitis should prompt a discussion with the oncologist and an early initiation of cortisone treatment.

In conclusion, immune checkpoint inhibitors can lead to a variety of different atypical pulmonary response patterns and adverse reactions. A knowledge of a patient’s clinical information helps to correctly interpret these changes and to support treatment decision-making.
